# Measurements of tendon length changes during stretch–shortening cycles in rat soleus

**DOI:** 10.1038/s41598-023-32370-5

**Published:** 2023-04-03

**Authors:** Atsuki Fukutani, Satoru Hashizume, Tadao Isaka

**Affiliations:** Faculty of Sport and Health Science, Ritsumeikan University, 1-1-1 Noji-higashi, Kusatsu, Shiga 525-8577 Japan

**Keywords:** Physiology, Medical research

## Abstract

The muscle force attained during concentric contractions is augmented by a preceding eccentric contraction (the stretch–shortening cycle (SSC) effect). At present, tendon elongation is considered the primary mechanism. However, we recently found that the magnitude of the SSC effect was not different, even after removing the Achilles tendon. To resolve these discrepant results, direct measurement of changes in Achille tendon length is required. Therefore, this study aimed to elucidate the influence of tendon elongation on the SSC effect by directly measuring the changes in Achilles tendon length. The rat soleus was subjected to pure concentric contractions (pure shortening trials) and concentric contractions with a preceding eccentric contraction (SSC trials). During these contractions, the Achilles tendon length was visualized using a video camera. The muscle force attained during the concentric contraction phase in the SSC trial was significantly larger than that in the pure shortening trial (*p* = 0.022), indicating the existence of the SSC effect. However, the changes in Achilles tendon length were not different between trials (i.e., the magnitude of tendon shortening attained during the shortening phase was 0.20 ± 0.14 mm for the SSC trial vs. 0.17 ± 0.09 mm for the pure shortening trial), indicating that the observed SSC effect is difficult to be explained by the elastic energy stored in tendons or muscle–tendon interaction. In conclusion, the effect of tendon elongation on the SSC effect should be reconsidered, and other factors may contribute to the SSC effect.

Dynamic movements, such as jumping or running, are composed of eccentric and concentric contractions. When concentric contractions are conducted immediately after an eccentric contraction, the concentric force is increased. This maneuver is known as the stretch–shortening cycle (SSC)^1–3^. In particular, the muscle force attained during concentric contractions is increased due to prior eccentric contractions^4,5^. Because this phenomenon is widely observed in human and animal movements, many studies have been conducted to elucidate the mechanisms of this force potentiation (i.e., the SSC effect). Based on the results of these studies, the currently suggested mechanisms are the stretch reflex^6,7^, tendon elongation (stored elastic energy and/or muscle–tendon interaction)^8–10^, preactivation^11–13^, and residual force enhancement^14–16^.

While other several factors have been proposed^17^, at present, tendon elongation is thought to be the most important factor affecting the SSC effect^10,18^. In particular, once the tendon is elongated during the prior eccentric contraction phase, the elongated tendon stores elastic energy, and this stored elastic energy can be released during the subsequent concentric contraction phase, leading to a larger mechanical work ^19^. In addition to the effect of elastic energy stored in the tendon, tendon length changes also lead to the optimization of muscle fascicle behavior, which is called muscle–tendon interaction^20–23^. When the tendon is elongated and shortened during SSCs instead of the muscle, the magnitude of muscle fascicle shortening during SSCs decreases. Because muscle force decreases when the shortening velocities increase based on the force–velocity relationship^24^, the decreased muscle fascicle shortening velocity due to tendon length changes also contributes to a larger muscle force^10,25^. Based on these two mechanisms, tendon elongation is widely considered the primary contributor to the SSC effect.

However, it is important to note that tendon length changes have not been measured directly in most previous studies^10,25^. For example, some studies measured muscle fascicle length changes and estimated muscle–tendon length changes from joint angle changes, and tendon length changes were calculated by subtracting the muscle fascicle length changes from the estimated muscle–tendon length changes^9,10,26^. However, we recently found that tendon length changes estimated by the above method were larger when the joint torque was smaller,that is, tendon length changes calculated during isometric twitch contractions were larger in the plantar flexion position than in the dorsiflexion position, whereas the twitch torque was larger in the dorsiflexion position than in the plantar flexion position^27^ (this interpretation is essentially the same even if the moment arm is considered). If this finding is correct, the above-estimated method requires caution because the magnitude of tendon elongation cannot be explained as a simple elastic body (not vary as a function of torque (force), unlike an elasticity). This phenomenon violates the simple elastic concept, Hooke's law. Furthermore, we also found that the magnitude of the SSC effect attained in the rat soleus muscle did not decrease when the Achilles tendon (free tendon) was removed^28^, indicating that the effect of tendon elongation on the SSC effect is negligible. Direct measurements of tendon length changes during SSCs are needed to explain the reason for these discrepant results.

Therefore, the purpose of this study was to measure Achilles tendon length changes directly during SSC and pure shortening contraction conditions. To achieve this, an in-situ rat model was adopted, and the soleus and Achilles tendons were isolated from other tissues. Achilles tendon length changes were visualized using a stereo microscope or video camera with a high spatial resolution (approximately 3 μm for the stereo microscope and 15 μm for the video camera). It was hypothesized that tendon length changes were very small based on our previous finding that the magnitude of the SSC effect was not different even after eliminating the free tendon^28^. To overcome this problem, we performed two experiments in this study,one was for recording the tendon elongation very precisely in the limited field of view with a stereomicroscope and the other one was for recording the tendon length change during SSCs in a wider field of view with a video camera.

## Materials and methods

### Muscle sample preparation and experimental setup

Nine samples from five male Sprague–Dawley rats (age, 20 weeks, body mass, 483 ± 40 g) were used in this study. The rats were fed standard rat chow and provided with water ad libitum. The rats were anesthetized with a gas mixture (2%–5% of isoflurane and O^2^) (KN-1071, Natsume Seisakusho, Japan) throughout the experiment. After the completion of all experimental trials, the rats were killed by an intraperitoneal injection of sodium pentobarbital (100 mg/kg of body mass). All procedures were approved by the Animal Care Committee of Ritsumeikan University, Biwako-Kusatsu Campus (BKC2017-036) and conducted according to the Guidelines for Proper Conduct of Animal Experiments (since June 1, 2006). All methods are reported in accordance with ARRIVE guidelines. The soleus muscle was used for all experiments because of its predominant composition of slow twitch fibers (84%)^29^, which minimized the effects of muscle fatigue. The soleus was exposed and dissected from its surrounding connective tissues, and its blood and nerve supplies left intact. Contractions were evoked using a specially designed nerve cuff electrode that was directly attached to the sciatic nerve. The gastrocnemius and plantaris muscles were cut to isolate the soleus tendon from a remnant piece of the calcaneus bone. The calcaneus was rigidly attached to a force transducer (UTA-500GR, Minebea Mitsumi, Japan) with a motor (DRSM42RG-04B2AZAK, Oriental Motor, Japan) using a grip (SC-8 IMADA, Japan) (Fig. 3). The two drills from the lateral sides were used to fix the tibia, and a grip (SC-8 IMADA, Japan) was also used to clamp the lower side of the tibia. To place the marker that can visualize the tendon length changes, a knot was made on the surface of the muscle–tendon sample using a silk suture. The tendon length was defined as the distance from the distal end of the Achilles tendon (end of the grip) to the silk knot (Fig. 3). A 0.9% physiological saline solution was applied to the preparation at least every two minutes to keep the muscles and tendons moist. Rectal temperature was monitored to maintain the animals’ body temperature within 35–37 °C using an infrared heat lamp and heating pad (BWT-100A, BRC, Japan). Muscle contractions were evoked by electrical stimulation (SEN3401, Nihon Kohden, Japan) using square-wave pulses (500 μs duration, 100 Hz). The stimulation voltage was gradually increased until a further increase in the voltage did not result in an increase in the twitch force. The voltage at this point is known as the saturation voltage. The stimulation voltage adopted for all experiments was set at 1.2 times the saturation voltage to ensure the activation of all motor units. Then, the optimal length (i.e., the muscle–tendon complex length where the maximal twitch force was obtained) was determined by adjusting the muscle length by 0.5 mm step length change. The muscle–tendon complex length (soleus and Achilles tendon length) at the optimal length was 40.4 ± 2.7 mm (27.2 ± 2.2 for the proximal side from the silk knot (see Fig. 3) and 13.1 ± 2.0 for the distal side from the silk knot).

### Experimental design

**Figure 1:**
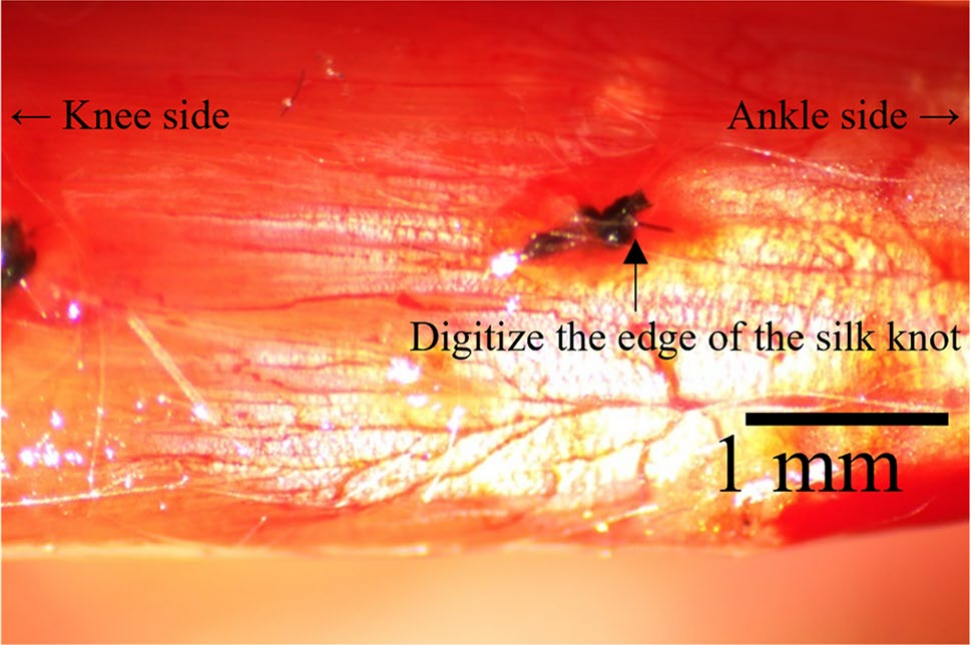
Typical image obtained by a microscope. The soleus and Achilles tendons were visualized under a microscope. The displacement of the knot tied near the end of the aponeurosis was recorded to examine tendon elongation during isometric contractions with high space resolution.

In the first experiment, isometric contraction with the maximal intensity was performed at the plateau region (optimal length), ascending limb (− 2 mm from the optimal length corresponding to about 5% of the muscle–tendon complex length), and descending limb (2 mm from the optimal length) in a random order. The duration of these contractions was 1.5 s, and the interval between the contractions was at least 2 min. During these contractions, the location of the silk knot was visualized using a stereomicroscope (SM-1TSW2-L6W-M, AmScope, US) equipped with a camera (HC210R, AmScope, US) (Fig. 1). In this experimental setting, the field of view was approximately 6 mm, and the number of pixels was 1824. Thus, the pixel resolution was approximately 3.3 μm. The sampling frequency was about 20 Hz.

**Figure 2:**
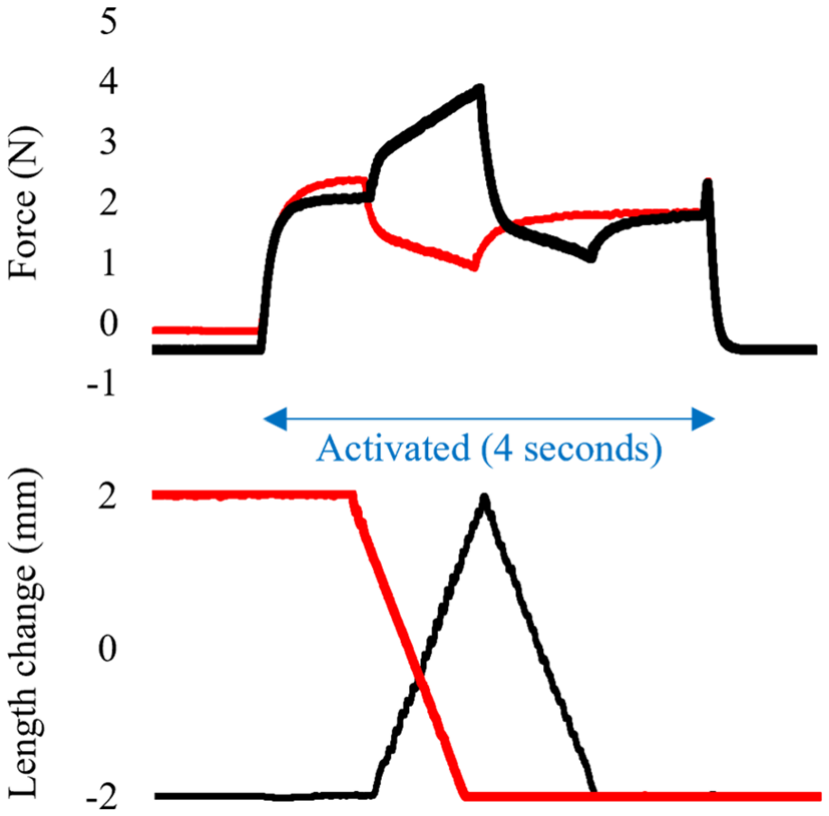
A representative sample of muscle force (upper panel) and muscle length (lower panel) as a function of time. The red line shows a trial of a concentric contraction with isometric preactivation (pure shortening condition) and the black line shows a trial of a concentric contraction with eccentric preactivation (SSC condition).

**Figure 3:**
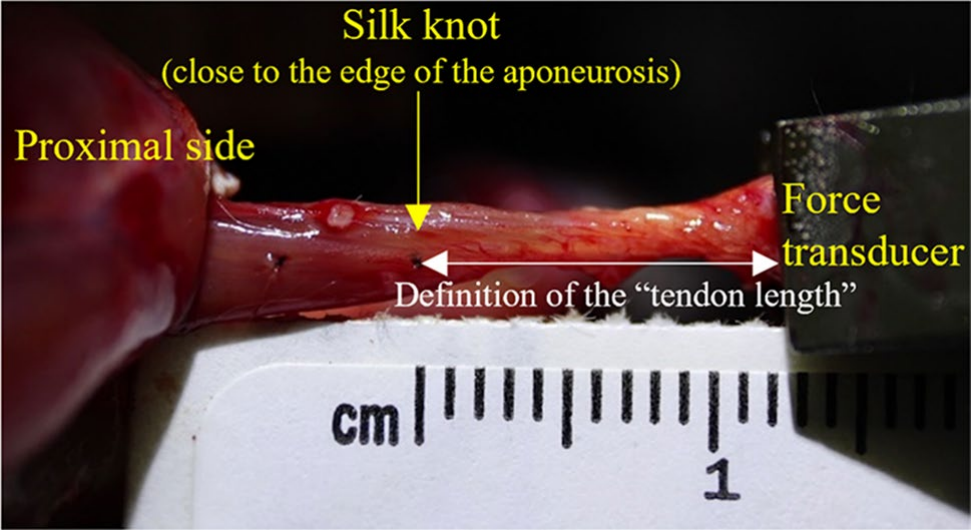
The typical image obtained by a video camera. The soleus and Achilles tendons were visualized using a video camera. The position of the silk knot and the edge of the black grip (force transducer) were recorded to measure the Achilles tendon length during the SSC and pure shortening trials.

In the second experiment, SSC and pure-shortening trials were conducted. For the SSC trial, isometric contractions were evoked at − 2 mm from the optimal length. The muscle–tendon complex was then actively stretched to 2 mm from the optimal length in 1 s. Immediately after the end of the stretch, the muscle–tendon complex was actively shortened to − 2 mm in 1 s, followed by isometric contraction for one second (Fig. 2). For the pure shortening trial, isometric contraction was evoked at 2 mm, and then, a muscle–tendon complex was actively shortened to − 2 mm in 1 s followed by isometric contraction for two seconds (Fig. 2). In addition to these active trials, passive trials, i.e., the identical length changes without activation, were performed to calculate the active force, i.e., the total force subtracted by the passive force. The contraction intensity of these two trials was maximal, and the contraction duration of these two trials was four seconds. The order of these trials was randomized, and the interval between contractions was at least 2 min. During these contractions, the location of the silk knot was visualized using a video camera (EOS R5; Canon, Japan) (Fig. 3). In this experimental setting, the field of view was approximately 60 mm, and the number of pixels was 4096. Thus, the pixel resolution was approximately 15 μm. The sampling frequency was 30 Hz.

### Measurements and data analyses

Muscle force and length were recorded at 1000 Hz using data acquisition software (Powerlab, ADInstruments, Japan). In the first experiment, the isometric force was calculated by subtracting the passive force from the total force attained at the same muscle length. Changes in tendon length due to muscle contraction were calculated from the displacement of the silk knot recorded by a stereomicroscope. This calculation was made from the images attained before the contractions and attained during the stable isometric phase. In the second experiment, the mean force attained during the entire shortening phase was calculated to evaluate the magnitude of the SSC effect^28^. In addition, the isometric force attained after the end of shortening was calculated. Changes in tendon length attained during the stretching phase and shortening phases were calculated from the displacement of the silk knot and distal end of the Achilles tendon (i.e., edge of the grip) (Fig. 3) recorded by the video camera. These calculations were made by obtaining the tendon length at the onset of the stretch, at the onset of the shortening, and at the end of the shortening. The tendon length changes attained during the stretching phase (the active stretch for the SSC trial and the passive stretch for the pure shortening trial because there was no active stretching phase) and the following shortening phase (for the SSC and pure shortening trials) were calculated.

### Statistics

For the first experiment, one-way analysis of variance (ANOVA) with repeated measures was conducted for isometric force and tendon elongation. When the main effect was significant, a following post-hoc test (Tukey’s HSD) was conducted. For the second experiment, a paired two-tailed t-test was conducted for the mean force attained during the shortening phase, tendon elongation attained during the stretching phase, and tendon shortening attained during the shortening phase between the SSC and pure shortening trials. The effect size for the ANOVA was calculated as partial η^2^, and that for the t-test was calculated as Cohen’s d. We performed the Kolmogorov–Smirnov test for all valuables (1: the isometric force for the first experiment, 2: the tendon elongation for the first experiment, 3: the mean force for the second experiment, 4–5: tendon elongation or shortening for the second experiment) and only the tendon elongation for the first experiment showed the significance. Thus, we also performed a non-parametric test (Friedman's test) for this variable and showed both parametric and non-parametric results. Statistical analyses were performed using SPSS (version 27, IBM, Japan), with the level of significance set at α = 0.05. All values are shown as means ± standard deviation.

## Results

**Figure 4:**
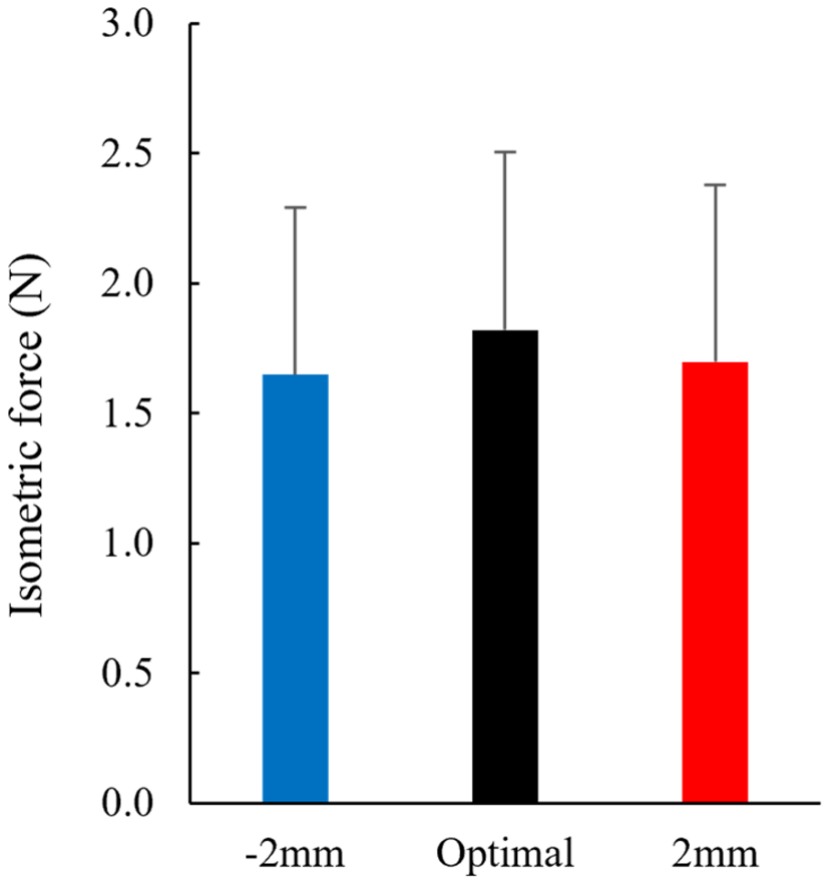
Comparison of the isometric force among − 2 mm, optimal, and 2 mm conditions (N = 9) (means ± SD).

**Figure 5:**
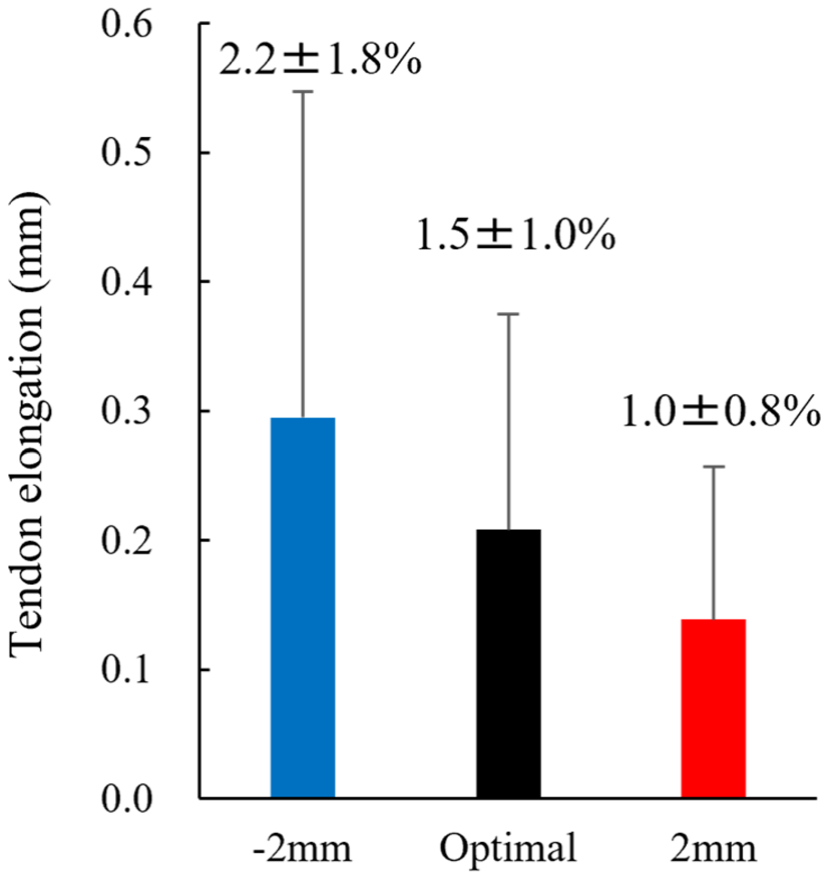
Comparison of the tendon elongation attained among − 2 mm, optimal, and 2 mm conditions (N = 9) (means ± SD). The values of strain (the magnitude of tendon elongation with respect to the entire tendon length, means ± SD) were added in the bar graph.

For the first experiment, the one-way ANOVA revealed that the isometric forces attained at the optimal length (1.82 ± 0.69 N), 2 mm (1.70 ± 0.68 N), and − 2 mm (1.65 ± 0.64 N) did not differ significantly (*p* = 0.088, partial η^2^ = 0.262), while the mean value was the largest in the optimal length (Fig. 4). The magnitude of tendon elongation obtained in the above three contractions was 0.21 ± 0.17 mm for the optimal length, 0.14 ± 0.12 mm for the 2 mm, and 0.29 ± 0.25 mm for the − 2 mm. While the one-way ANOVA revealed that these values were significantly different (*p* = 0.005, partial η^2^ = 0.530), the post-hoc test indicated no significant differences between the conditions (*p* = 0.191–0.643) (Fig. 5). We also performed the non-parametric test because the Kolmogorov–Smirnov test showed significance. Friedman's test showed a significant difference among conditions and the post hoc test showed a significant difference between − 2 and 2 mm conditions (*p* = 0.005).

**Figure 6:**
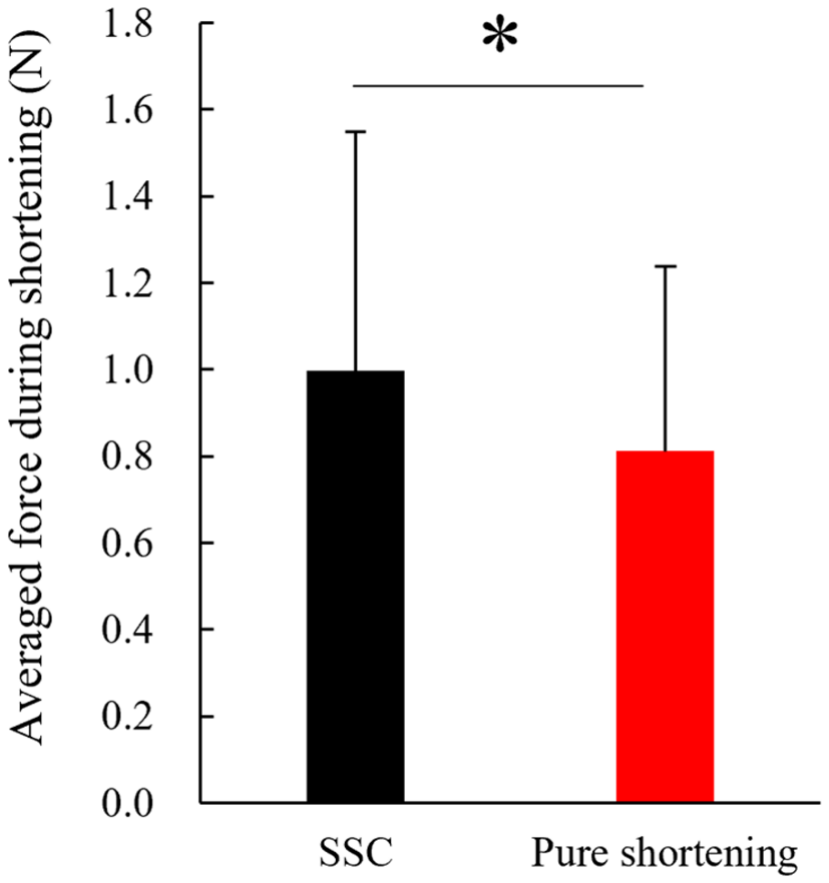
Comparison of the SSC effect attained between SSC and pure shortening conditions (N = 9) (means ± SD). * indicates the significant difference between conditions.

**Figure 7:**
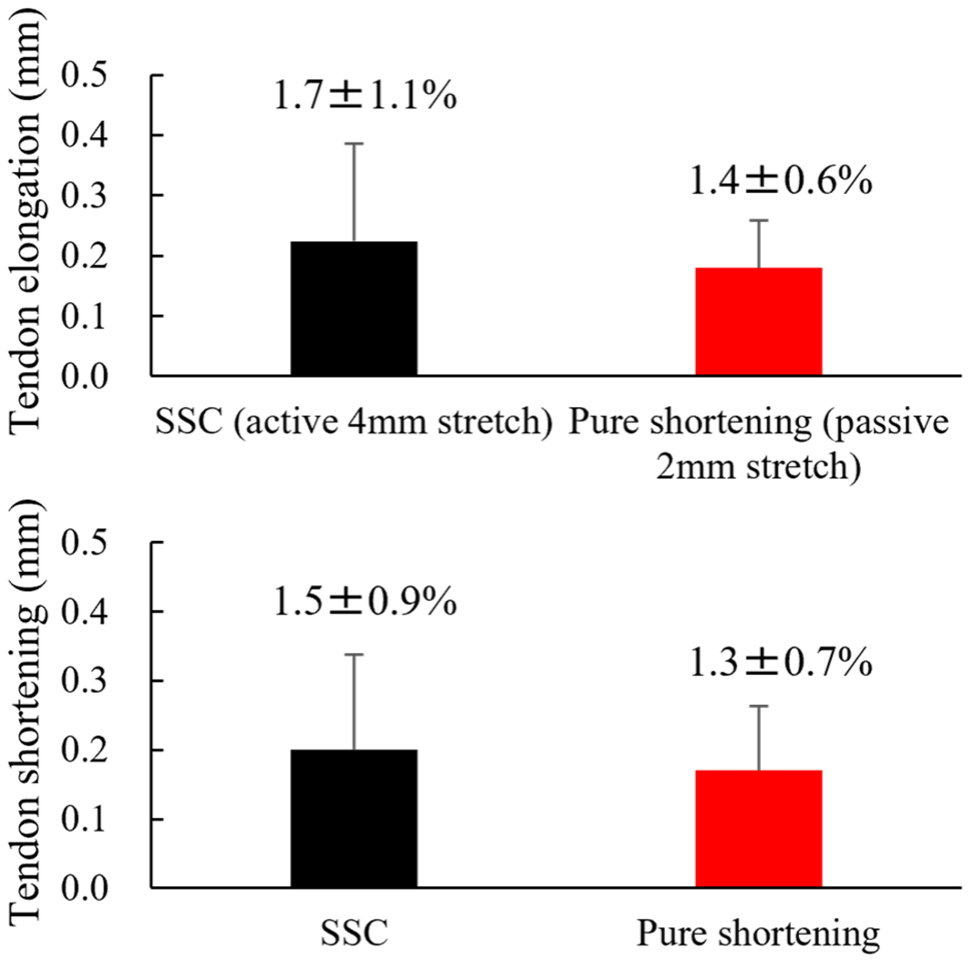
Comparison of the tendon elongation attained during the stretching phase (upper panel) and the tendon shortening attained during the shortening phase (lower panel) between SSC and pure shortening conditions (N = 9) (means ± SD). The values of relative length changes (the magnitude of tendon length changes with respect to the entire tendon length, means ± SD) were added in the bar graph. Note that the elongation conditions were different as described in the upper graph (stretched actively from − 2 to 2 mm for the SSC, while stretched passively from 0 to 2 mm for the Pure shortening).

For the second experiment, the paired t-test revealed that the mean force attained during the shortening phase was significantly larger in the SSC (1.00 ± 0.55 N) than in the pure shortening trial (0.81 ± 0.43 N) (*p* = 0.022, Cohen’s d = 0.948) (Fig. 6). On the other hand, the magnitude of tendon elongation attained during stretch (active stretch for the SSC trial and passive stretch for the pure shortening trial) was not different significantly between SSC (0.22 ± 0.16 mm) and pure shortening trial (0.18 ± 0.08 mm) (*p* = 0.312, Cohen’s d = 0.13). Similarly, the magnitude of tendon shortening attained during the pure shortening phase was not different significantly between SSC (0.20 ± 0.14 mm) and pure shortening trial (0.17 ± 0.09 mm) (*p* = 0.322, Cohen’s d = 0.09) (Fig. 7).

## Discussion

In this study, changes in Achilles tendon length were measured directly to examine the contribution of tendon elongation to the SSC effect. In our experimental setup, the force attained during the shortening phase was larger in the SSC trial than in the pure shortening trial, indicating that the SSC effect was successfully induced. However, the changes in Achilles tendon length did not differ between the trials. This result indicated that tendon elongation could not explain the observed SSC effect, at least in this experimental setting. This finding is similar to our previous study, which reported that the magnitude of the SSC effect did not decrease even after eliminating the Achilles tendon^28^.

A possible reason for the negligible contribution of tendon elongation to the SSC effect is the magnitude of the tendon elongation. The observed tendon elongation and shortening attained in the SSC trial was 0.22 ± 0.16 mm and 0.20 ± 0.14 mm, respectively. Considering the tendon length (13.13 ± 2.04 mm), the strain of tendon elongation attained during the stretching phase in the SSC trial was 1.7 ± 1.1%. Because the magnitude of muscle–tendon complex elongation during the stretching phase was 4 mm, the Achilles tendon contributed to 3.8 ± 0.2% of muscle–tendon elongation, which seems to be too small to predict the contribution of elastic energy stored in the tendon or muscle–tendon interaction to the SSC effect. In addition, the magnitude of tendon elongation attained between the SSC and pure shortening trials was not significantly different. This result seems strange because the magnitude of tendon elongation was not a function of the force applied to the elastic body, the Achilles tendon. The most plausible explanation for no significant difference between the larger force condition (SSC trial) and the smaller force condition (pure shortening trial) is that the magnitude of tendon elongation is very small, so these small force differences did not induce the substantial tendon length difference. Related to this point, we also measured the magnitude of Achilles tendon elongation attained during isometric contraction with a better space resolution (approximately 3.3 μm/pixel), and found that (although the statistical difference was not observed, based on the raw values) the magnitude of tendon elongation was larger at the − 2 mm condition while the isometric force was smaller. Although no statistical difference of observed, the tendon elongation attained in the optimal length condition was always smaller than that attained in the − 2 mm condition, and the isometric force attained in the optimal length condition was always larger than that attained in the − 2 mm condition. Because the magnitude of tendon elongation is not a function of force, we cannot consider the Achilles tendon as a series elastic component. If so, the magnitude of tendon elongation does not necessarily represent the magnitude of elastic energy stored in the Achilles tendon. These results are essentially similar to those of our previous study using human plantar flexor muscles, which reported that the magnitude of tendon elongation estimated by the widely used method, that is, subtracting the measured muscle fascicle length changes from the estimated muscle–tendon complex length changes based on joint angle changes^9,26,30^, was smaller in the dorsiflexed position, while the joint torque was larger compared to the anatomical or plantarflexed position^27^. One possible reason for these strange results is the slack of a muscle–tendon complex. As discussed in a previous study^27^, if tissues have a slack, the tissues can be lengthened (evaluated by the straight distance between both ends) without storing elastic energy. This concept can consistently explain our experimental findings because the magnitude of tendon elongation was larger in the shortened position and smaller in the stretched position^27^, current study). Therefore, most of the tendon elongation observed in our − 2 mm condition (short muscle–tendon complex length condition) could be derived from eliminating the slack of the muscle–tendon complex.

In addition to the elastic energy stored in tendons, muscle–tendon interactions have also been considered as the mechanism of the SSC effect^9,26,30^. However, it is difficult to explain the observed SSC effect in our study by muscle–tendon interaction because the magnitude of tendon shortening attained during the shortening phase was very small (0.20 ± 0.14 mm, corresponding to 3.8 ± 0.2% of the shortening in total), and no significant difference in tendon shortening was observed between the SSC and pure shortening trials (0.17 ± 0.09 mm). Therefore, in this experimental setting, the observed SSC effect should be derived from mechanisms other than tendon elongation. Because muscle contractions were induced by identical electrical stimulation parameters between the SSC and pure shortening trials, the stretch reflex should not contribute to the observed SSC effect. In addition, at least 1 s of isometric preactivation was conducted before the SSC or pure shortening maneuver, indicating that the effect of preactivation should be negligible. Therefore, other mechanisms, such as cross-bridge kinetics or residual force enhancement, would be the primary contributors to the observed SSC effect^17^.

Several previous studies have performed direct tendon length measurements similar to our current study. Finni et al.^31^ and Maas et al.^32^ examined the tendon length change during isometric contraction with different joint angles and reported that the elongation of the tendon differed among plantar flexors. The reported displacement and strain of the tendon were larger than those reported in our study. They measured the displacement and the resultant strain from the coordinates of 4 markers (within the 5 mm range based on their Fig. 1) attached to the Achilles tendon. Thus, the actual whole tendon elongation in an absolute manner would be larger. In addition, although we do not know the reason, the tendon of the soleus was shortened when they activated the gastrocnemius (the soleus was not activated) (their Fig. 5). According to their explanation, the rotation of the tendon due to the activation would affect their measurement values. If so, there is a possibility that the influence of this rotation was also included in other measurement values, which affects the reported displacement/strain values.

In our experimental setting, the effect of tendon elongation on SSC was negligible. However, this result can vary depending on the situation. First, we used the rat soleus and Achilles tendons. The Achilles tendon of rats is attached to not only the soleus but also the gastrocnemius, which is much stronger than the soleus. Therefore, the force applied to the Achilles tendon during everyday activities in rats could be larger than that adopted in this study. In such cases, tendon elongation can be substantial and consequently contribute to the SSC effect. In addition, even if the strain of the tendon is small (e.g., 1.7% in this study), tendons can contribute to the SSC effect from the perspective of muscle–tendon interaction. If the ratio of muscle length to tendon length is 1:1, a 1% change in tendon length corresponds to a 1% change in muscle length, which seems that the influence of muscle–tendon interaction is small. However, if the ratio of muscle length to tendon length is 1:10, a 1% change in tendon length corresponds to a 10% change in muscle length. If the magnitude of muscle elongation or shortening changes by 10%, it is likely that the force-generating capability of the muscle can be changed substantially due to force–length and force–velocity relationships^24,33^. Regarding this point, some previous studies reporting the significant contribution of tendons to the SSC effect were adopted to kangaroos or turkeys^25,34^, which would have a relatively longer tendon with respect to muscle length. These species have a positive effect on tendon elongation based on the ratio of muscle length to tendon length. Thus, care should be taken to expand the results obtained from other species.

As a limitation, our measurements included not only free tendon but also aponeurosis. Thus, there is a possibility that if the aponeurosis “shortened” during the contractions, the actual free tendon elongation was underestimated. However, based on the images recorded in this study, it was highly unlikely that the aponeurosis shortened during contractions. In addition, even if the aponeurosis behaves differentially with respect to the free tendon, the influence of this would be small because the length of aponeurosis included in our measurements (about 1–2 mm) was much shorter than that of the free tendon (about 11–12 mm). Finally, the number of samples is not so large (N = 9 from 5 rats). However, our main finding that the magnitude of tendon elongation was small in SSCs should not be changed by increasing the sample size.

## Conclusions

In conclusion, the contribution of Achilles tendon elongation to the SSC effect was negligible, at least in the case of the rat soleus and Achilles tendon. This is likely due to the small amount of tendon elongation and shortening attained during SSCs. Based on our findings, tendon elongation does not necessarily explain the SSC effect, and factors other than tendon elongation can contribute to the SSC effect. The effect of tendon elongation on the SSC effect should be affected by the geometry of the tendon, which differs among species. Therefore, the geometric information of the tendon should be measured directly to discuss the influence of the tendon in detail.

## Data Availability

The datasets used and/or analyzed during the current study are available from the corresponding author on reasonable request.
